# Clinical features of asthma with comorbid bronchiectasis: A systematic review and meta-analysis

**DOI:** 10.1097/MD.0000000000023858

**Published:** 2021-01-29

**Authors:** Shi-Qi Zhang, Xiao-Feng Xiong, Zuo-Hong Wu, Ting-Ting Huang, De-Yun Cheng

**Affiliations:** Department of Respiratory and Critical Care Medicine, West China Hospital, Sichuan University, Chengdu, China.

**Keywords:** comorbidity, eosinophils, high-resolution computed tomography, lung function, smoking

## Abstract

**Background:**

This meta-analysis aimed to systematically estimate the prevalence of comorbid bronchiectasis in patients with asthma and to summarize its clinical impact.

**Methods:**

Embase, PubMed, and Cochrane Library electronic databases were searched to identify relevant studies published from inception until March 2020.

**Study Selection:**

Studies were included if bronchiectasis was identified by high-resolution computed tomography. Outcomes included the prevalence of bronchiectasis and its association with demographic characteristics and indicators of asthma severity, including results of lung function tests and the number of exacerbations.

**Results:**

Five observational studies with 839 patients were included. Overall, the mean prevalence of bronchiectasis in patients with asthma was 36.6% (307/839). Patients with comorbid bronchiectasis had lower forced expiratory volume in 1 second (FEV_1_)/forced vital capacity (FVC) (MD: −2.71; 95% CI: −3.72 to −1.69) and more frequent exacerbations (MD: 0.68; 95% CI: 0.03 to 1.33) than those with asthma alone, and there was no significant difference of sex, duration of asthma and serum levels of immunoglobulin(Ig)Es between asthmatic patients with or without bronchiectasis.

**Conclusion:**

The presence of bronchiectasis in patients with asthma was associated with greater asthma severity. There are important therapeutic implications of identifying bronchiectasis in asthmatic patients.

## 1 Introduction

Asthma is a widespread and heterogeneous disease that currently afflicts 300 million people worldwide.^[1,2]^ It is characterized by chronic airway inflammation that leads to respiratory symptoms, including cough, wheezing, chest tightness, and dyspnea. In the 2019 statement of the Global Initiative for Asthma,^[3]^ it was emphasized that the primary goal of optimal asthma management is the recognition and management of comorbidities, including those that can manifest with similar symptoms to asthma. The identification of these comorbidities is of fundamental importance in distinguishing between patients with true refractory asthma and those patients who are difficult to treat due to comorbidities or complications.

Bronchiectasis (BE) is a progressive disease characterized by irreversible enlargement of the bronchi, which leads to increased secretion production and airway obstruction.^[4,5]^ With increased usage of computed tomography (CT), the presence of bronchiectasis in patients with asthma is being identified more frequently, particularly in patients with severe asthma.^[6]^ The etiological relationship between the two conditions has not yet been precisely established. It is necessary to recognize the overlapping symptomatology in patients with comorbid bronchiectasis and asthma because it may change patient management strategies, especially in consideration of increased frequency of exacerbations and greater disease severity.

Hence, the present meta-analysis aimed to summarize the current findings on the comorbidity of bronchiectasis and asthma, to clarify the clinical impact of bronchiectasis on patients with asthma, and to examine whether this comorbidity contributed to asthma severity.

## 2 Methods

The protocol for this study is registered on the PROSPERO register of systematic reviews. All analyses were based on data from previously published studies and, therefore, this study did not require ethical approval or patient consent.

### 2.1 Search strategy for identification of studies

An electronic literature search was performed to identify clinical studies that investigated the impact of bronchiectasis on patients with asthma. The Embase, PubMed, and the Cochrane Library databases were comprehensively searched to identify all relevant clinical studies in human beings published from inception until March 10, 2020 using the following search terms: “asthma” and “bronchiectasis”. Publication species was limited to humans and language was limited to English. In addition, relevant review articles and their reference lists were checked manually.

### 2.2 Study selection

Manuscripts were included if they met the following criteria:

(1) asthma was defined according to the guidelines of the Global Initiative for Asthma (GINA) or another standard diagnostic definition;
(2) bronchiectasis was confirmed by high-resolution CT (HRCT);
(3) one or more demographic or clinicopathologic characteristics, including age, sex, smoking history, serum levels of IgEs and eosinophils, lung function, duration of asthma, and frequency of exacerbations, were compared in patients with asthma with and without comorbid bronchiectasis.

Studies were excluded if they met the following criteria:

(1) reported as conference abstracts, case reports, editorials, or narrative reviews;
(2) the presence of bronchiectasis was assessed by chest X-ray only; or
(3) only included occupational or single sex subjects.

Two investigators (SQZ and XFX) independently obtained the full-text versions of potentially eligible manuscripts and screened all references according to the selection criteria. Any discrepancies were resolved through discussion.

### 2.3 Data extraction and quality assessment

Two investigators (SQZ and XFX) independently extracted the following data from the selected studies: the first author's name; year of publication; patients’ age, sex, and smoking history; levels of IgE and eosinophils in serum; duration of asthma, postbronchodilator forced expiratory volume(FEV1)% predicted, postbronchodilator ratio of FEV1/forced vital capacity(FVC); and frequency of exacerbations in the previous year. The quality of included studies was evaluated according to the Agency for Healthcare Research and Quality standard. Any disagreement was resolved through discussion, or adjudicated by a third author (ZHW).

### 2.4 Data synthesis and analysis

The meta-analysis was conducted using the open-source Review Manager (RevMan) software (Version 5.3.4), available at the following website: (http://ims.cochrane.org/revman/download). The relationships between clinical features, including sex, smoking history and presence of atopy, were assessed using odds ratios (ORs) and 95% confidence intervals (95% CI). Other outcomes were assessed using mean differences (MD) and 95% CI. Random-effects models were utilized when there was heterogeneity (I^2^ > 50%); otherwise, fixed-effects models were used.^[7]^ Heterogeneity was quantified using a visual forest plot inspection with the I^2^ statistic and the χ^2^ test. Sensitivity analyses were carried out to further explore heterogeneity based on characteristics of the study.

## 3 Results

### 3.1 Description of included studies

**Figure 1:**
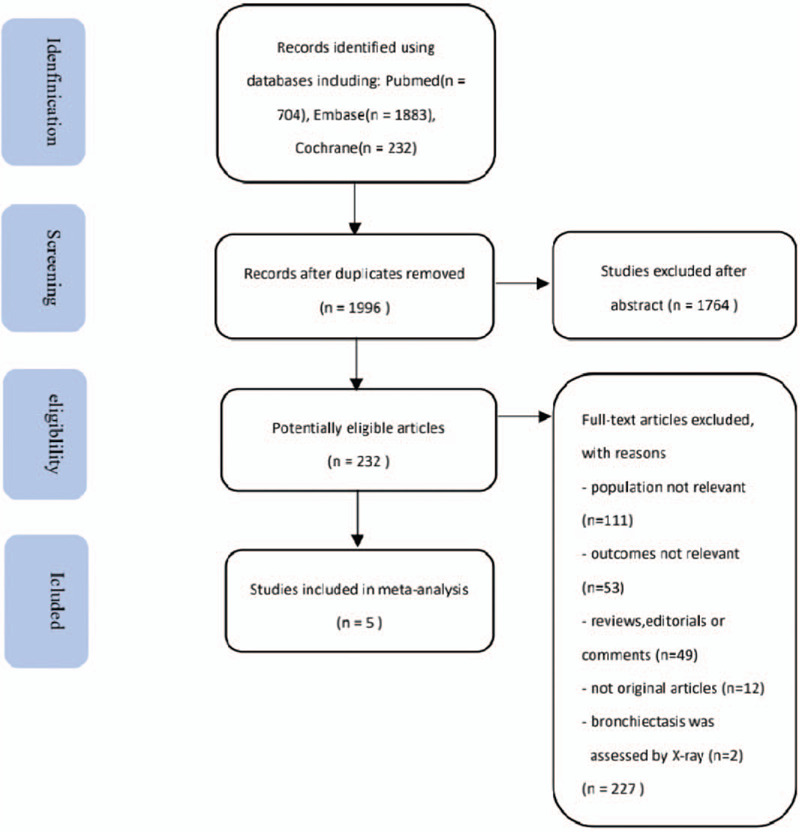
Flow chart of identification of studies include.

Based on electronic database searches, 1996 potentially relevant articles were identified. After reviewing abstracts, 232 articles were found to be eligible for further evaluation. Of these, 227 articles were excluded because the population was not relevant (n = 111), outcomes were not relevant (n = 53), studies were reviews, editorials or comments (n = 49), no original articles (n = 12), or bronchiectasis was assessed by X-ray (n = 2). Thus, a total of five studies met the inclusion criteria (Fig. 1).

### 3.2 Study characteristics

Five observational studies with 839 patients with asthma were included in the meta-analysis, and 307 (36.6%) of the patients in these studies had comorbid bronchiectasis. The number of participants per study ranged from 37 to 398, and there were no study restrictions based on age or sex. Two of the included studies employed a prospective design.^[8,9]^ HRCT was used to diagnose bronchiectasis in all included studies.

Outcome reporting varied among the studies. Age and lung function were reported in all included studies, serum IgE levels were reported in four studies,^[8,10–12]^ smoking history were reported in 3 studies,^[10–12]^ duration of asthma was reported in three studies,^[8,12,13]^ serum eosinophil levels were reported in 2 studies,^[10,11]^ and frequencies of asthma exacerbations were reported in 3 studies.^[10–12]^ The characteristics of all included studies are summarized in Tables 1 and 2. Risk of bias summary of included studies is shown in Table 3.

**Table 1:** Characteristics of studies included in this meta-analysis.

Study	Authors (year, country)	Duration	Study disign	Severity of asthma	Sources of patients
1	Takemura et al^[8]^ (2004, Japan)	Two months	Prospective cohort study	Not mentioned	Clinic
2	Menzies et al^[13]^ (2011, England)	Not mentioned	Retrospective study	Severe	Clinic
3	Padilla-Galo et al^[11]^ (2018, Spain)	Three years	Prospective study	Moderate, severe	Hospital
4	Coman et al^[10]^ (2018, Spain)	2010-2013	Retrospective study	Severe	Clinic
5	Garcia-Clemente et al^[12]^ (2019, Spain)	2015-2017	Retrospective study	Severe	Hospital

**Table 2:** Radiological characteristics of asthma patients in the studies included.

Study	Authors (year, country)	CT slice thickness	Number (bronchiectasis/Total asthma)	Diagnostic criteria of bronchiectasis
1	Takemura et al^[8]^ (2004, Japan)	3 mm collimation at 20 mm intervals	23/37	Bronchial lumen was larger than the cross-section of the accompanying pulmonary artery
2	Menzies et al^[13]^ (2011, England)	5 mm collimation at 20 mm intervals	47/133	Not mentioned
3	Padilla-Galo et al^[11]^ (2018, Spain)	Not mentioned	113/398	Method of Martinez-Garcia et al.^[33]^
4	Coman et al^[10]^ (2018, Spain)	Not mentioned	86/184	(1) Absence of bronchus tapering in the periphery of the lungs, (2) bronchus with an internal diameter larger than that of its accompanying vessel
5	Garcia-Clemente et al^[12]^ (2019, Spain)	Not mentioned	38/87	According to the Spanish Society of Pneumology and Thoracic Surgery(SEPAR) recommendations^[33]^

**Table 3:** Risk of bias summary of included studies.

Agency for Healthcare Research and Quality standard					
for cross-sectional study quality	Takemura et al, 2004^[8]^	Menzies et al,2011^[13]^	Padilla-Galo et al, 2018^[11]^	Coman et al, 2018^[10]^	Garcia-Clemente et al, 2019^[12]^
Define the source of information	Yes	Yes	Yes	Yes	Yes
List inclusion and exclusion criteria for exposed and unexposed subjects (cases and controls) or refer to previous publications	Yes	Yes	Yes	Yes	Yes
Indicate time period used for identifying patients	No	Yes	Yes	Yes	Yes
Indicate whether or not subjects were consecutive if not population based	Yes	Yes	Yes	Yes	Yes
Indicate if evaluators of subjective components of study were masked to other aspects of the status of the participants	No	No	Yes	Yes	Yes
Describe any assessments undertaken for quality assurance purposes	Yes	Yes	Yes	Yes	Yes
Explain any patient exclusions from analysis	Yes	Yes	Yes	Yes	Yes
Describe how confounding was assessed and/or controlled	Yes	Yes	Yes	No	Yes
If applicable, explain how missing data were handled in the analysis	No	No	No	No	No
Summarize patient response rates and completeness of data collection	Yes	Yes	No	Yes	Yes
Clarify what follow-up, if any, was expected and the percentage of patients for which incomplete data or follow-up was obtained	Yes	No	Yes	No	No

### 3.3 Clinical features

**Figure 2:**
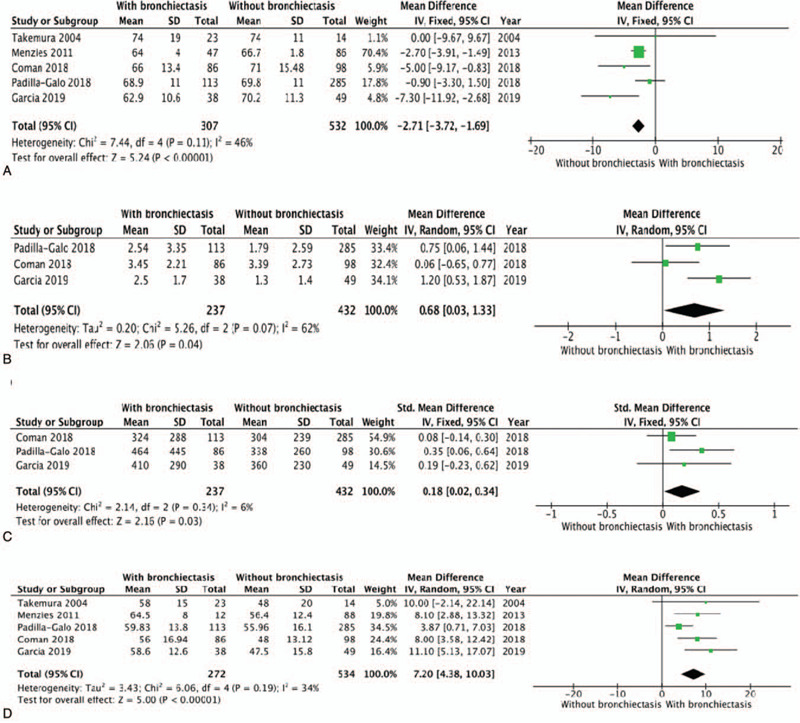
Forest plots of study. A. Forest plots of postbronchodilator FEV1/FVC%; B. Forest plots of exacerbations; C. Forest plots of serum eosinophils; D. Forest plots of age. CI = confidence interval, IV = inverse variance, M-H = Mantel-Haenszel method, SD = standard deviation.

**Figure 3:**
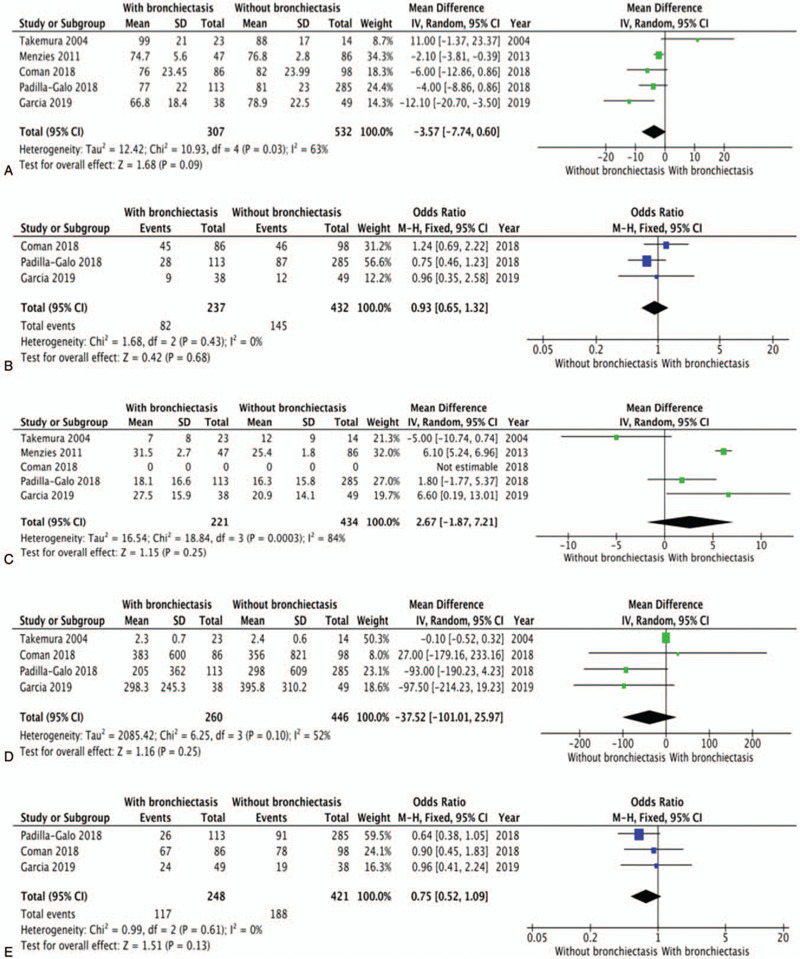
Forest plots of study(Forest plot of postbronchodilator FEV_1_% predicted; Forest plot of smoking history; Forest plot of duration of asthma; Forest plot of serum IgE; Forest plot of sex). CI = confidence interval, IV = inverse variance, M-H = Mantel-Haenszel method, SD = standard deviation.

Meta-analysis of the 5 studies shows that airway obstruction was more severe in patients with comorbid asthma and bronchiectasis, those patients showed a lower postbronchodilator FEV_1_/FVC ratio (MD: -2.71; 95% CI: -3.72 to -1.69; *P* < .00001; Fig. 2A) when compared with patients with asthma alone. But there was no significant difference in FEV1/predicted between two groups as mentioned above (Fig. 3A). The included studies used the number of exacerbations of asthma in the previous year to indicate the severity of the patient's disease, the incidence of asthma exacerbations in previous year was higher in patients with asthma and bronchiectasis than in patients without bronchiectasis (MD: 0.68; 95% CI: 0.03 to 1.33; *P* = .04; Fig. 2B).

### 3.4 Allergy biomarkers

Blood eosinophil levels were higher in patients with comorbid asthma and bronchiectasis compared with those without bronchiectasis (MD: 0.18; 95% CI: 0.02 to 0.34; *P* = .03; Fig. 2C). In other hand, the impact on serum IgE levels can not be found when compared patients with comorbid asthma and bronchiectasis with patients without bronchiectasis.

### 3.5 Demographic characteristics

Meta-analysis of these studies showed that patients with comorbid asthma and bronchiectasis were older than those without bronchiectasis (MD: 7.20; 95% CI: 4.38 to 10.03; *P* < .00001; Fig. 2D). But the related studies showed no significant different in the sex, smoking history and duration of asthma between patients with comorbid asthma and bronchiectasis and those with asthma alone.

### 3.6 Sensitivity analysis

**Figure 4:**
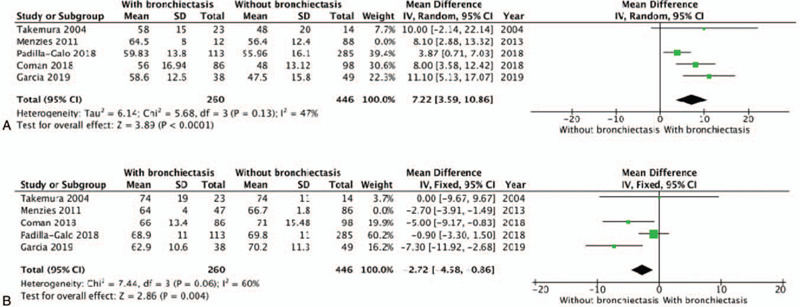
Sensitivity analysis of study (Sensitivity analysis of results on FEV1/FVC; Sensitivity analysis of results on age).

In the studies by Menzies et al,^[13]^ the included population did not exclude patients with bronchiectasis with known causes (e.g., Allergic bronchopulmonary aspergillpsis, etc.), which might have contributed to confounding interference in the included population in this meta-analysis. Since this study had some proportion influence in the meta-analysis, sensitivity analyses was carried out for the five studies excluding of the study by Menzies et al.^[13]^ The sensitivity analyses showed that the meta-analysis results related to age, lung function, and number of asthma exacerbations did not change (Fig. 4).

## 4 Discussion

A growing body of evidence suggests that asthma is not only a functional airway disease, but is also a structural disorder, with the development of bronchiectasis frequently considered a consequence of long-term uncontrolled asthma.^[14]^ To our knowledge, this meta-analysis is the first to pool published data to estimate the prevalence of bronchiectasis in patients with asthma and to summarize its complications. The presence of bronchiectasis in patients with asthma varied from 2% to 80% in reported studies.^[15,16]^ In the present report, it was shown that coexistence of bronchiectasis and asthma is not rare, is more frequently identified in older patients, and may be associated with a smoking history. This finding is consistent with the consensus that smoking is one of the most important causes of bronchiectasis.^[5]^

The cause and effect relationship between bronchiectasis and asthma is still debatable, mainly due to the lack of relevant animal studies.^[17]^ It is hypothesized, however, that the two abnormalities share several inflammatory mechanisms that may influence each other. For example, the neutrophilic airway inflammation in patients with asthma may induce epithelial damage and defective mucociliary clearance may result in further airway injury and remodeling.^[18–20]^ Moreover, imbalances between proteases (mainly neutrophil elastase) and antiproteases may lead to lung tissue destruction.^[21,22]^ At the same time, recurrent airway infection and increased bronchial secretions in patients with bronchiectasis may contribute to airway obstruction and airflow limitation, which can lead to exacerbations of underlying asthma or increased asthma symptoms.^[23]^ The persistent chronic inflammatory processes of asthma and bronchiectasis are further aggravated by frequent exacerbations.^[24]^

The most significant finding of this meta-analysis was the increased exacerbation rate of patients with comorbid asthma and bronchiectasis, potentially indicating that these patients are more poorly controlled in comparison to patients with asthma alone. This finding may be explained by several potential effects of the comorbid bronchiectasis. Coexistent bronchiectasis is associated with severe airflow obstruction, and it is well known that lung function decline is associated with a higher incidence of exacerbations. Also, bronchiectasis is a complex chronic disease, with readily identifiable airway remodeling and mucus hypersecretion with subsequent formation of mucous plugs, which are also known risk factors for asthma exacerbations. Third, persistent bacterial colonization of the airways in bronchiectasis leads to the neutrophil-dominated inflammation and a vicious cycle of active neutrophil elastase and proteases.^[18,25]^ Studies have provided evidence that the use of antibiotics can reduce the density of bacteria, the presence of bronchial inflammation, and the frequency of exacerbations in individuals with bronchiectasis, as well as in individuals with asthma.^[26,27]^ Regrettably, only a few studies have described chronic potentially pathogenic microorganisms (PPMs) colonization data, and the related data were too limited to carry out a meta-analysis.

Although this meta-analysis showed no significant difference in asthma duration between the two groups, in large part of known studies indicate that the time from clinical diagnosis of asthma in the patients with comorbid bronchiectasis was longer than in patients without bronchiectasis. First, it may support the perspective that bronchiectasis is a consequence of asthma and, therefore, needs time to develop.^[28]^ Also, it may indicate that the clinical features of bronchiectasis in patients with asthma may be overshadowed by the symptoms of asthma.

The hypothesis that asthma and atopy may have a role in the pathogenesis of bronchiectasis was first postulated in 1939. Since then, some studies have reported a direct link between bronchiectasis and bronchial hyperreactivity or atopy.^[29,30]^ In our study, however, we did not identify a positive relationship between bronchiectasis and atopy. In addition, the higher level of serum eosinophils we observed in patients with coexistent bronchiectasis suggests that the presence of bronchiectasis could potentially be used to identify a phenotype of patients with severe eosinophilic asthma (SEA).^[31]^

As our study demonstrates, the presence of bronchiectasis is more than just a radiological finding. It may also have a real impact on the natural history of a patient's asthma and may have important prognostic value in the evaluation and management of this disease. Previously, a scoring system [NOPES(after Fe**NO**, **p**neumonia, **e**xpectoration and **s**everity) score] to assess the risk of bronchiectasis in uncontrolled moderate-to-severe asthmatic patients was proposed in a study by Padilla-Galo et al,^[11]^ with a specificity of 95%. This scoring system was based on the concept that bronchiectasis is related to the severity of asthma, chronic expectoration, a history of pneumonia, and lower levels of Fractional exhaled nitric oxide (FeNO). And this scoring system may be implemented in patients with asthma to calculate the probability of bronchiectasis.

Guidline of asthma recommended steroid therapy as the gold standard for severe asthma.^[3]^ On the contrary, inhaled steroids were not recommended in bronchiectasis because of higher bacterial loads in airway and bronchiectasis is often associated with chronic bacterial infections. According to the studies on patients with bronchiectasis, the use of inhaled steroids appears to be associated with an increase risk of infections and hospitalizations, even if it is not clearly related to an increase of mortality.^[32]^ But this dose not affect therapeutic strategy of asthma and COPD.^[3,33]^ It is therefore appropriate to add the treatment of bronchiectasis to the standard therapy of asthma for a better control of infections and a better management of the secretions, such as longterm antibiotic therapy and airway clearance.^[34]^ Recently, mepolizumab-a anti-IL-5 biologic drug-was widely used for the treatment of patients with severe asthma and bronchiectasis, it is demonstrated that this drug can reduce exacerbations of asthma and bronchiectasis by effects on eosinophilic inflammation.^[35]^ Further researches on eosinophils in the pathogenesis of patients with asthma and bronchiectasis as well as the efficacy of an anti-eosinophilic therapy are needed.

Our meta-analysis had several limitations. First, patients in some studies were not graded for the severity of their asthma and, thus, the precise incidence of bronchiectasis in patients with different severity levels of asthma could not be calculated. Second, only two of the included studies had a prospective cohort design,^[8,9]^ accordingly, limitations in the methodological quality of the included studies can be identified. Finally, due to lack of data, meta-analyses of health-related quality of life (QoL), NSAID hypersensitivity, treatment and mortality were not performed.

Despite these limitations, the available data were still sufficient to perform a reliable meta-analysis with important therapeutic implications in patients with bronchiectasis and asthma. This mata-analysis showed that recognition of comorbid of bronchiectasis in patients with asthma is important for treatment and follow-up decisions, especially in consideration of disease severity. To avoid the “inadequate treatment” or “excessive therapy”,^[36]^ a CT scan of the thorax to identify potential bronchiectasis should be performed in patients with asthma who have symptoms of airway infection or a poor response to conventional treatments.

## 5 Conclusion

This meta-analysis provides further evidence of the association between bronchiectasis and severity of asthma. Moreover, it shows that the presence of bronchiectasis in patients with asthma is associated with severe pulmonary insufficiency, higher risk of exacerbations, and a worse prognosis. From the clinical point of view, we conclude that this subgroup of patients may need tailer treatment targeting chronic mucus production and airway bacterial infection, in addition to the recommended treatments for patients with asthma, in order to improve symptoms and to reduce future risks. Moreover, we conclude that more prospective cohort studies should be conducted to identify whether coexistent bronchiectasis should be considered a pathological phenotype of asthma, which may have important predictive value in clinical care.

## Acknowledgments

We are grateful to the authors of the primary studies included in this meta-analysis.

## Author contributions

**Data curation:** Xiaofeng Xiong, Zuohong Wu, Tingting Huang.

**Formal analysis:** Xiaofeng Xiong, Tingting Huang.

**Investigation:** Zuohong Wu.

**Methodology:** Zuohong Wu.

**Supervision:** Deyun Cheng.

**Visualization:** Tingting Huang.

**Writing – original draft:** Shiqi Zhang.

**Writing – review & editing:** Shiqi Zhang, Deyun Cheng.

## Abbreviations

Abbreviations: COPD = chronic obstructive pulmonary disease, CT = computed tomography, FE_NO_ = fractional exhaled nitric oxide, FEV1 = forced expiratory volume in 1 second, FVC = forced vital capacity, HRCT = high-resolution CT, IgE = immunoglobulin E.
